# Problem Behavior in Autism Spectrum Disorder: Considering Core Symptom Severity and Accompanying Sleep Disturbance

**DOI:** 10.3389/fpsyt.2019.00487

**Published:** 2019-07-12

**Authors:** Ebony Lindor, Carmel Sivaratnam, Tamara May, Nicole Stefanac, Katherine Howells, Nicole Rinehart

**Affiliations:** Faculty of Health, School of Psychology, Deakin Child Study Centre, Deakin University, Geelong, VIC, Australia

**Keywords:** autism spectrum disorder, symptom severity, sleep, accompanying disturbance, problem behavior

## Abstract

In addition to the core symptoms that define autism spectrum disorder (ASD), many individuals experience broader problem behavior at a level significant enough for families to seek further clinical assessment and intervention. We define “problem behavior” as any significant emotional or behavioral issue captured by the Child Behavior Checklist (CBCL) including anxiety, depression, withdrawal, somatic complaints, problems with socialization, thought or attention, rule-breaking, and aggression. While greater ASD symptom severity and accompanying sleep disturbance have each been linked with more severe problem behavior, there is little understanding about how these two key factors interact; that is, it is unclear whether the severity and type of sleep disturbance an individual experiences differentially influences the relationship between ASD symptom severity and problem behavior. The aim of the current study was, thus, to explore whether the link between greater ASD symptom severity and clinically elevated problem behavior is moderated by the presence/degree of accompanying sleep disturbance. Forty males with ASD, aged 5–12, participated in the study. The Social Responsiveness Scale, CBCL, and Children’s Sleep Habits Questionnaire were administered to obtain information about ASD symptom severity, problem behavior, and sleep habits, respectively. Results indicated that the relationship between ASD symptom severity and problem behavior differed among individuals with ASD depending on the degree of sleep disturbance they experienced. Specifically, there was a significant positive relationship between ASD symptom severity and problem behavior for individuals with no sleep disturbance or milder sleep disturbance (i.e., in these cases, individuals with severe ASD symptoms experienced clinically elevated problem behavior, while those with milder ASD symptoms experienced milder problem behavior). In contrast, there was no significant relationship between ASD symptom severity and problem behavior for individuals with moderate-to-severe sleep disturbance; rather, clinically significant problem behavior was apparent across all individuals irrespective of ASD symptom severity. Follow-up analyses indicated that disturbances in sleep duration, disordered breathing, and daytime sleepiness were related to clinically elevated problem behavior even among those with milder ASD symptoms. These findings emphasize the importance of routinely assessing for accompanying sleep disturbance in this population regardless of whether individuals present with mild, moderate, or severe ASD.

## Introduction

Autism spectrum disorder (ASD) is a heterogenous neurodevelopmental disorder characterized by social deficits and restricted, repetitive patterns of behavior (1). Core symptoms typically manifest as poor social-emotional reciprocity, abnormal non-verbal communication, difficulty developing and maintaining social relationships, repetitive movements, intense fixations/interests, and atypical sensory sensitivity. In addition, a significant proportion of individuals with ASD exhibit a broader range of problem behaviors that extend beyond the core symptoms that define the disorder (2). For example, there is existing research to suggest that many individuals with ASD experience clinically significant internalizing and externalizing problems including anxiety, depression, somatization, rule-breaking, aggression, self-harm, inattention, hyperactivity, impulsivity, and abnormal thought (3–9). In this paper, the term “problem behavior” encompasses any significant emotional or behavioral issue captured by the Child Behavior Checklist (CBCL) including problems with anxiety, depression, withdrawal, somatization, socialization, thought, attention, rule-breaking, and aggression. As is common in ASD research, the nature and severity of these broader problem behaviors vary considerably among individuals on the spectrum; there are some individuals who experience relatively few of these problem behaviors, while many others exhibit one or more problem behaviors that are significant enough to warrant clinical concern (3, 10, 11) and may even result in comorbid diagnoses such as anxiety, depression, conduct disorder, oppositional defiant disorder (ODD), and/or attention deficit hyperactivity disorder (ADHD). Being able to predict who on the spectrum (i.e., individuals of which severities and clinical presentations) is more likely to exhibit clinically significant problem behavior is particularly important, as those with persistent difficulties in this area are at greater risk of long-term mental health issues, poor academic achievement, and future crime and violence (12, 13).

Researchers have, thus, become increasingly interested in studying the factors related to clinically elevated problem behavior in different individuals with ASD. While a range of factors have been examined in the literature, the relevance and contribution of accompanying sleep disturbance have emerged as an area of particular interest (14, 15). Sleep plays an important role in memory consolidation (16) and, more generally, brain plasticity, which in turn supports typical cognitive, emotional, and behavioral development (17). Sleep problems are prevalent in ASD and reportedly affect 40% to 80% of children; these estimates are significantly greater than that of the typically developing population and place those who are already developmentally vulnerable at a greater disadvantage (18, 19). In ASD, the exact etiology of accompanying sleep disturbance is unclear; however, researchers commonly argue that sleep problems are contributed to by a complex combination of biological, psychological, and social/environmental factors (18). While neurophysiological and neurochemical abnormalities can promote chronic sleep–wake disturbance and abnormal timing of melatonin secretion in this population, comorbid medical conditions and current medications also need to be considered, as do other potential factors such as core ASD symptoms, child-rearing practices, and family stress (14, 18, 20). In ASD, common types of sleep disturbance include delayed sleep latency, reduced sleep efficiency, decreased total sleep duration, poor sleep maintenance/night waking, bedtime resistance, and daytime sleepiness (14, 21). Improving our understanding of how the nature and severity of these common sleep problems vary among individuals with ASD may help us conceptualize the possible consequences they can have on the behavior and broader functioning of these individuals. Given that sleep disturbance has been found to be amenable to intervention, there are also tangible benefits to focusing our efforts on sleep in ASD.

In terms of existing research, there is evidence to suggest that sleep disturbance in ASD may exacerbate problem behavior. For example, poor sleep has been linked to increased internalizing and externalizing problems, including tantrums, oppositional behavior, physical aggression, irritability, self-injury, depression, anxiety, mood variability, inattention, and hyperactivity (15, 22–30). Recently, Cohen et al. (31) found direct evidence that greater variation in sleep timing and duration can predict subsequent problematic daytime behavior (e.g., aggression, self-injury, tantrums, and property destruction), and there is also longitudinal evidence to suggest that sleep problems may predict later anxiety (32). Sleep researchers frequently acknowledge that, although highly prevalent, sleep problems do not affect all individuals with ASD. As such, a number of studies have categorized ASD participants as “poor” sleepers and “good” sleepers, based on actigraphy, polysomnography, and parent-report measures, to examine behavioral differences among those with and without significant sleep problems. A large-scale study conducted by Goldman et al. (25) indicated that poor sleepers with ASD exhibited a significantly higher percentage of problem behavior than did good sleepers with ASD. Researchers relying on parent-report measures have revealed that individuals with ASD and accompanying sleep disturbance demonstrate greater externalizing and internalizing problem behaviors than do those without accompanying sleep disturbance (27, 29). Others who have taken advantage of objective measures have demonstrated similar relationships; for example, Malow et al. (33) reported more significant affective and social problems in individuals identified as poor sleepers, while Goldman et al. (23) noted greater levels of inattention, hyperactivity, and restricted, repetitive behavior.

The severity of sleep disturbance in ASD and the particular type of sleep disturbance that individuals experience have also been identified as important considerations. In terms of severity, Adam et al. (29) revealed different behavioral profiles in those with mild versus severe sleep problems, whereby individuals with more severe sleep problems exhibited significantly greater externalizing and overall problem behavior; surprisingly, internalizing behaviors were not sensitive to sleep severity in this study. This is somewhat in contrast to other researchers who have found both significantly greater internalizing and externalizing problems in individuals with moderate-to-severe sleep disturbance relative to those with milder sleep disturbance (28). In terms of identifying particularly relevant types of sleep disturbance, Hirata et al. (19) reported that insomnia was strongly associated with problem behavior; Mazurek and Sohl (15) identified night wakings as having the most consistent link with behavior problems, and Fadini et al. (34) noted relationships between behavioral problems and disorders of arousal (e.g., sleepwalking, sleep terrors, and nightmares) and excessive somnolence (e.g., difficulties rising and daytime sleepiness). These studies highlight the importance of considering the severity and type of accompanying sleep disturbance when attempting to understand problem behavior in ASD.

While the above research outlines that both the presence and severity of sleep problems may be importantly linked to problem behavior in ASD, there is broader research to suggest that ASD symptom severity is another key factor to consider. Notably, there is accumulating research to suggest a strong positive association between ASD symptom severity and problem behavior. Researchers have indicated that those with more severe ASD symptoms are likely to experience significantly more problem behaviors that are also of greater severity than those with moderate or mild ASD symptoms (24, 35–37). There is also longitudinal evidence to suggest that ASD symptom severity is a key predictor of later emotional and behavioral problems in children and adolescents with high-functioning ASD (38). Studies such as these emphasize that ASD severity itself is related to increased problem behavior. Complicating the picture, there is also evidence to suggest that more severe ASD symptoms are associated with more significant sleep disturbance (23, 39–41). While the research in this area is sound and it is often the case that sleep problems are more pronounced in individuals with severe ASD presentations, it is important to note that sleep problems do not exclusively present in this subgroup and can affect individuals across the full spectrum (14).

Although there is a large body of evidence indicating that ASD severity and accompanying sleep disturbance are important factors each associated with more severe problem behavior in ASD, it remains unclear whether the presence/severity of an accompanying sleep disturbance differentially influences the relationship between ASD severity and problem behavior. In other words, more research is required to understand whether the positive relationship between ASD symptom severity and problem behavior (which suggests that those with greater ASD symptoms exhibit clinically elevated problem behavior while those with milder ASD symptoms exhibit significantly milder problem behavior) changes depending on the degree and type of sleep disturbance an individual experiences. The current study, therefore, aimed to examine whether the presence/degree of accompanying sleep disturbance moderates the relationship between ASD severity and problem behavior and to further explore the relevance of different types of sleep disturbance. While we expected ASD severity and degree of sleep disturbance to each show an association with problem behavior, there was limited literature available on which to base a hypothesis on how these two key factors would interact. Although somewhat exploratory from this perspective, the findings from this study were expected to improve our ability to identify those on the spectrum who may be more likely to experience clinically significant problem behavior.

## Materials and Methods

### Participants

We recruited 40 children with ASD to the study *via* a range of methods. Advertisement flyers were distributed to community institutions, networking websites, and social media (e.g., Autism Victoria, Deakin Child Study Centre Facebook, early intervention services, and private pediatric clinics). There were also a number of primary schools and special development schools who expressed interest in supporting the study and agreed to send letters of invitation to families in their community containing advertising information about the study. Because this particular study was contained within a broader longitudinal project that aimed to promote the inclusion of children of all abilities into physical activity, the NAB AFL Auskick database was also used as an avenue of recruitment.

For inclusion in the study, participants were required to have a pre-existing formal diagnosis of ASD. To meet formal diagnosis in Victoria, Australia, an individual must satisfy DSM criteria, have undergone assessment by a multidisciplinary panel (e.g., medical, psychology, and speech clinicians), and had the diagnosis confirmed by a pediatrician or child psychiatrist. Participants were required to be aged between 5 and 12 and attend primary school (or equivalent special education). The intention was to capture a broad range of functioning levels; as such, no specific requirement was enforced around level of functioning or comorbidities. Testing locations were offered throughout metropolitan and regional Melbourne, which permitted a broad, representative community sample.

### Measures

#### Demographics and Level of Functioning

Basic demographic information including age, gender, and handedness was obtained through parent report. Level of adaptive functioning was measured using the Vineland Adaptive Behavior Scale—Third Edition (VABS-3) (42). The domain-level parent/caregiver form was selected for use in this study; it is appropriate for ages 3 to 90+ and consists of three core domains (i.e., communication, daily living, and social skills and relationships), each of which contains 40 items. An overall adaptive behavior composite score was calculated from the 120 core items (*M* = 100, *SD* = 15). Scores between 86 and 114 reflect “adequate” adaptive level. Scores between 115 and 129 can be considered “moderately high,” and those above 130 as “high.” In comparison, scores from 71 to 85 can be considered “moderately low” and those at or below 70 as “low.” In terms of psychometric properties, this form has an internal consistency coefficient of .97 and test–retest reliability of .87. Validity has previously been established and is further supported by intercorrelation data, assessment against other measure of adaptive functioning, and specific standardization for clinical samples [see Ref. (42)].

#### The Social Responsiveness Scale—Second Edition (SRS-2) School Age Form

The SRS-2 was administered to measure ASD symptom severity (43). The school age form consists of 65 items relevant to children aged 4 to 18, which measures five core areas of functioning: social awareness, social cognition, social communication, social motivation, and restricted interests and repetitive behavior. Using a four-point Likert scale (i.e., 1 = not true, 2 = sometimes true, 3 = often true, and 4 = almost always true), parents were required to select the degree to which each of the items applied to their child in the past 6 months. Parent ratings were summed to obtain an SRS-2 total score (*M* = 50, *SD* = 10). Total scores at or below 59 are considered to be within normal limits; total scores placed between 60 and 65 indicate mild deficiencies in reciprocal social behavior; scores between 66 and 75 suggest moderate deficiencies; and those at 76 or above reflect severe deficiencies in reciprocal social behavior.

#### The Children’s Sleep Habits Questionnaire (CSHQ)

The CSHQ was administered to measure a range of behaviorally based and medically based sleep problems (44). It consists of 33 core items that relate to eight sleep domains: bedtime resistance, sleep onset delay, sleep duration, sleep anxiety, night waking, parasomnias, sleep-disordered breathing, and daytime sleepiness. Parents were required to rate each of the items on the basis of a typical week using a three-point Likert scale (i.e., 1 = usually, defined as occurring five to seven times per week; 2 = sometimes, defined as occurring two to four times per week; and 1 = rarely, defined as occurring zero to one time per week). Normative data (i.e., means and standard deviations) are available for each of the domains, from which *T*-scores can be calculated for ease of interpretation. Generally, *T*-scores between 60 and 64 are considered higher than average and may indicate slightly more concerns than is typical; those between 65 and 69 are notably elevated and border clinical significance, while those above 70 warrant clinical concern. For total sleep problems, a cutoff point of 41 has been established; scores over 41 indicate clinically significant sleep problems, while scores below 41 reflect sleep behavior within normal limits (44). With the use of this cutoff point, psychometric properties are acceptable, with a sensitivity estimate of .80 and a specificity estimate of .72. Assigning severity to total sleep problems has been inconsistently performed across studies. Of note, however, a previous large-scale study (i.e., *N* > 1,000) described scores from 41 up to 55 as mild to moderate and scores above 56 as moderate to severe, which were obtained by calculating the 75th percentile/fourth quartile (28). While all variables in this study were analyzed in continuous form, these descriptive categories were adopted to describe variability among individuals where significant interactions were revealed (i.e., to interpret the “pick a point” output from simple slopes analyses and zones of significance produced by the Johnson–Neyman technique at a clinically meaningful level; see Data Analysis section for further details).

#### The Child Behavior Checklist (CBCL)

The CBCL was administered to measure problem behavior. Given that primary-school-aged children were recruited to the study, two forms were required to capture the age ranges, the 1.5–5-year-old form (45) and the 6–18-year-old form (46). The 1.5–5-year-old form consists of 100 age-relevant items, whereas the 6–18-year-old form consists of 113 age-relevant items. Parents were required to rate the degree to which each of the items applied to their child over the past 6 months using a three-point Likert scale (i.e., 0 = not true, 1 = somewhat/sometimes true, and 2 = very/often true). Each of these forms summarizes child emotional and behavioral problems into measures of internalizing problems, externalizing problems, and total problems. The total problem *T*-score was used for the purposes of this study with *T*-scores at or above 70 taken to reflect “clinically significant” problem behavior. Given the broad range of emotional and behavioral problems captured by the CBCL, it should be noted that individuals with problems at this level may meet criteria for comorbid diagnoses such as anxiety, depression, conduct disorder, ODD, and/or ADHD. In terms of psychometrics, the CBCL is a widely used well-validated measure that has been adequately shown to discriminate between clinical and non-clinical behavioral problems (87% accuracy for school-aged forms; 84% accuracy for pre-school form).

### Procedure

Ethics approval was obtained through Deakin University Human Research Ethics Committee and the Victorian Department of Education and Training. In accordance with the Declaration of Helsinki, parents formally provided informed consent, while children gave verbal assent. Following the consent process, parents were asked to complete a brief survey consisting of demographic questions (e.g., date of birth) and medical history (e.g., details of diagnoses and health services attended). They then completed the VABS-3 to provide information on their child’s level of functioning. Given that the data for this study were collected as part of a larger longitudinal project involving direct child measures, parents and children physically attended testing sessions. In most cases, parents completed the SRS-2, CSHQ, and CBCL during the session, while their child engaged in motor and cognitive tasks relevant to the broader project. If this was not possible (e.g., school-based testing sessions or insufficient time to complete measures), parents were permitted to complete the questionnaires in their own time and return them to the researchers, either at the initial session or after the session by reply-paid envelope. In cases where parents were unable to complete the questionnaires prior to or at the session, they were encouraged to complete the questionnaires and return by post at their earliest convenience.

### Data Analysis

To characterize the sample, descriptive statistics were conducted for 1) level of adaptive functioning (VABS-3), 2) ASD severity (SRS-2), 3) problem behavior (CBCL), and 4) sleep disturbance (CSHQ). Correlations among these variables were also conducted. A moderation analysis was then run to examine whether the relationship between ASD symptom severity and problem behavior varied as a function of the participants’ level of accompanying sleep disturbance. Follow-up moderation analyses were conducted to explore the most relevant types of sleep disturbance. All data were analyzed using IBM SPSS Statistics Version 21, with the assistance of the PROCESS macro (47). All variables were mean centered for analysis, and the HC3 (Davidson–MacKinnon) heteroscedasticity-consistent inference applied. Where moderation was found, the main analysis was followed by simple slopes analysis and then the Johnson–Neyman technique to identify the true zone of significance. Given the relatively small sample, a power analysis was conducted to ensure our approach for the main moderation analysis. Assuming the following parameters - large effect size, α error probability of .05, and power of .80 - a total sample size of 36 was required.

## Results

### Sample Characteristics and Correlations

The sample was exclusively male, and ages ranged from 5.02 to 12.87, with an *M* of 8.23 and *SD* of 2.12. **Table 1** includes detailed information on participant characteristics, including level of functioning (VABS-2), ASD severity (SRS-2), problem behavior (CBCL), and sleep (CSHQ). **Table 2** further displays correlations among these variables.

**Table 1:** Participant characteristics.

	M	SD	Range
Age Adaptive functioning ABC composite Communication Daily living Social skills ASD severity SRS-2 total Social awareness Social cognition Social communication Social motivation RRB index SCI index Problem behavior Internalizing Externalizing Total CBCL problems Sleep disturbance Bedtime resistance Sleep onset delay Sleep duration Sleep anxiety Night wakings Parasomnias Sleep-disordered breathing Daytime sleepiness Total CSHQ	8.23 72.98 75.85 74.75 72.35 76.93 73.40 73.53 75.18 68.75 77.70 75.55 63.93 61.78 66.75 57.65 62.58 59.53 60.88 62.75 67.60 53.98 56.90 48.18	2.12 8.85 12.02 11.95 10.17 9.30 10.41 10.77 10.51 10.71 8.68 9.56 9.89 11.43 9.38 15.41 16.88 19.33 16.38 18.97 19.38 17.99 10.08 9.68	5.02–12.87 54–89 46–105 52–95 49–91 56–90 48–90 53–90 51–90 48–90 53–90 54–90 41–82 33–83 46–83 44–97 45–83 46–110 44–99 44–100 41–113 46–141 44–91 36–72

All of the above, with the exception of age and total CSHQ, have been calculated based on T-scores. Total CSHQ is based on raw data with a clinical cutoff point of 41 (see Measures section for further information).

**Table 2:** Correlations.

	Age	ABC composite	SRS-2 Total	Total CBCL	Total CSHQ
Age	1								
ABC Composite	−.32*	1						
SRS-2 total	.04	−.54**	1				
Total CBCL	.06	−.12	.57**	1		
Total CSHQ	.01	−.19	.43**	.62**	1

**p < .01, *p < .05.

### Total Sleep Problems as a Moderator

The overall linear model revealed that ASD symptom severity (SRS-2 total score), total sleep problems (CHSQ total score), and their interaction significantly predicted level of problem behavior (CBCL total score), *F*(3, 36) = 10.90, *p* < .001, *R* ^2^ = .55. Individually, ASD symptom severity predicted level of problem behavior, *b* = .36, *t* = 2.90, *p* = .006 [.11, .62], as did total sleep problems, *b* = .48, *t* = 4.45, *p* = .001 [.26, .69]. Most importantly, the interaction between ASD symptom severity and total sleep problems bordered significance, *b* = −.02, *t* = −2.00, *p* = .05 [−.04, −.0003], indicating that the relationship between ASD symptom severity and problem behavior differs depending on the degree of accompanying sleep disturbance present.

**Figure 1:**
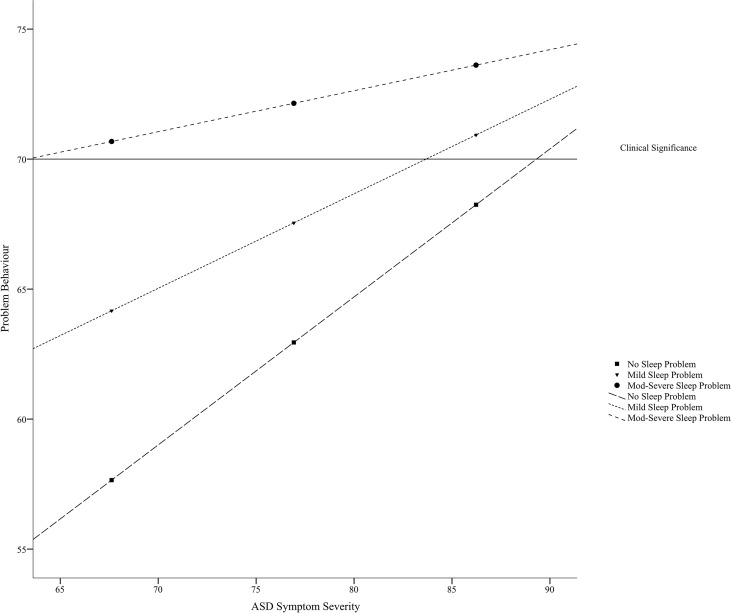
Relationship between ASD symptom severity (i.e., SRS-2 score) and problem behavior (i.e., total CBCL score) as moderated by degree of sleep disturbance (i.e., total CSHQ score).

A follow-up simple slopes analysis revealed that the relationship between ASD symptom severity and problem behavior was only significant for those in the sample with no sleep problems (i.e., estimated at a score of 38.50), *b* = .57, *t* = 3.04 *p* = .004 [.19, .95], or mild sleep problems (i.e., estimated at a score of 48.18), *b* = .36, *t* = 2.90, *p* = .006 [.11, .62]. For individuals with moderate-to-severe sleep problems (i.e., estimated at a score of 57.86), the relationship between ASD symptom severity and problem behavior was not significant, *b* = .16, *t* = 1.20, *p* = .24 [−.11, .43]; rather, the degree of problem behavior was high at all severities of ASD. See **Figure 1**. The Johnson–Neyman technique further clarified the zone of significance was from a total sleep score of 36.00 (lower bound of data) to 53.92. The relationship between ASD severity and problem behavior was no longer significant for individuals in the sample with total sleep scores above 53.92.

### Contribution of Specific Sleep Problems

**Table 3** summarizes the linear models examining the relationship between ASD symptom severity and problem behavior as moderated by each of the specific sleep domains: 1) bedtime resistance, 2) sleep onset delay, 3) sleep duration, 4) sleep anxiety, 5) night waking, 6) parasomnias, 7) disordered breathing, and 8) daytime sleepiness.

**Table 3:** Linear models summarizing the relationship between ASD symptom severity and problem behavior as moderated specific sleep disturbances.

	Model	*b*	SE	*t*	*p*	CI
1	Constant ASD severity Bedtime resistance ASD severity × bedtime resistance *Note.* *F*(3,36) = 12.60, *p* < .001, *R* ^2^ = .51	66.94 .50 .24 −.01	1.10 .13 .06 .01	61.08 3.83 3.82 −.90	<.001 <.001 <.001 .37	[64.72, 69.16] [.23, .76] [.11, .37] [−.02, .01]
2	Constant ASD severity Sleep onset delay ASD severity × sleep onset delay *Note.* *F*(3,36) = 7.89, *p* < .001, *R* ^2^ = .36	67.06 .63 −.09 −.01	1.41 .13 .09 .01	47.58 4.73 −1.04 −.48	<.001 <.001 .31 .63	[64.20, 69.92] [.36, .90] [−.26, .09] [−.03, .02]
3	Constant ASD severity Sleep duration ASD severity × sleep duration *Note. F*(3,36) = 9.32, *p* < .001, *R* ^2^ = .42	68.00 .35 .22 −.01	1.22 .13 .08 .01	55.66 2.62 2.90 −2.26	<.001 .01 .006 .03	[65.52, 70.48] [.08, .62] [.07, .38] [−.03, −.002]
4	Constant ASD severity Sleep anxiety ASD severity × sleep anxiety *Note. F*(3,36) = 8.54, *p* < .001, *R* ^2^ = .45	66.84 .54 .18 −.01	1.19 .13 .06 .01	56.40 4.10 2.86 −1.50	<.001 <.001 .007 .14	[64.43, 69.24] [.27, .81] [.05, .30] [−.02, .004]
5	Constant ASD severity Night waking ASD severity × night waking *Note. F*(3,36) = 11.44, *p* < .001, *R* ^2^ = .54	66.81 .50 .23 −.002	1.13 .16 .05 .01	59.22 3.21 4.42 −.28	<.001 .003 <.001 .78	[64.52, 69.10] [.18, .82] [.12, .34] [−.02, .01]
6	Constant ASD severity Parasomnias ASD severity × parasomnias *Note. F*(3,36) = 7.62, *p* < .001, *R* ^2^ = .48	67.27 .45 .20 −.01	1.25 .14 .08 .01	53.86 3.08 2.38 −1.12	<.001 .004 .02 .27	[64.74, 69.81] [.15, .74] [.03, .37] [−.03, .01]
7	Constant ASD severity Disordered breathing ASD severity × disordered breathing *Note. F*(3,36) = 9.32, *p* < .001, *R* ^2^ = .42	67.88 .51 .24 −.02	1.22 .13 .09 .01	55.48 3.89 2.69 −3.16	<.001 <.001 .01 .003	[65.39, 70.36] [.24, .77] [.06, .41] [−.04, −.01]
8	Constant ASD severity Daytime sleepiness ASD severity × daytime sleepiness *Note. F*(3,36) = 9.66, *p* < .001, *R* ^2^ = .43	67.60 .45 .31 −.03	1.28 .13 .15 .01	52.61 3.59 2.12 −2.46	<.001 .001 .04 .02	[65.00, 70.21] [.20, .71] [.01, .60] [−.05, −.005]

As is evident from **Table 3**, sleep duration, disordered breathing, and daytime sleepiness were found to significantly moderate the relationship between ASD symptom severity and problem behavior. To detail the nature of these interactions, the sections below outline the simple slopes analyses and Johnson–Neyman results.

#### Moderating Effect of Sleep Duration

Simple slopes analysis revealed that the relationship between ASD symptom severity and problem behavior was only significant for those who had no problems with sleep duration (i.e., estimated at a *T*-score of 46.00), *b* = .55, *t* = 3.21 *p* = .003 [.20, .89], or few problems with sleep duration (i.e., estimated at a *T*-score of 59.53), *b* = .35, *t* = 2.62, *p* = .01 [.08, .62]. For individuals who had clinically significant problems with sleep duration (i.e., estimated at a *T*-score of 78.86), the relationship between ASD symptom severity and problem behavior was not significant, *b* = .07, *t* = .43, *p* = .67 [−.27, .41]; rather, the degree of problem behavior was high at all severities of ASD. The Johnson–Neyman technique further clarified the zone of significance was from a *T*-score of 46.00 (lower bound of data) to a *T*-score of 65.15. The relationship between ASD symptom severity and problem behavior was no longer significant for individuals in the sample with a *T*-score above 65.15.

#### Moderating Effect of Disordered Breathing

A similar pattern of findings was evident for disordered breathing. Simple slopes analysis revealed that the relationship between ASD symptom severity and problem behavior was only significant for those who had no problems with disordered breathing (i.e., estimated at a *T*-score of 46.00), *b* = .68, *t* = 4.45, *p* <.001 [.37, .99], or very few problems with disordered breathing (i.e., estimated at a *T*-score of 53.97), *b* = .51, *t* = 3.89, *p* <.001 [.24, .77]. For individuals who had clinically significant problems with disordered breathing (i.e., estimated at a *T*-score of 71.96), the relationship between ASD symptom severity and problem behavior was not significant, *b* = .12, *t* = .77, *p* = .45 [−.20, .44]; again, the degree of problem behavior was high at all severities of ASD. The Johnson–Neyman technique further clarified that the zone of significance was from a *T*-score of 46.00 (lower bound of data) to a *T*-score of 64.90. The relationship between ASD symptom severity and problem behavior was not significant for individuals in the sample with a *T*-score above 64.90. It was, however, noted that the relationship became significant again for those with very high *T*-scores above 117.26.

#### Moderating Effect of Daytime Sleepiness

As per sleep duration and disordered breathing, simple slopes analysis revealed that the relationship between ASD symptom severity and problem behavior was only significant for those who had no problems with daytime sleepiness (i.e., estimated at a *T*-score of 46.82), *b* = .75, *t* = 4.59, *p* < .001 [.42, 1.08], or few problems with daytime sleepiness (i.e., estimated at a *T*-score of 56.90), *b* = .45, *t* = 3.59, *p* = .001 [.20, .71]. For individuals who had borderline clinically significant daytime sleepiness (i.e., estimated at a *T*-score of 66.98), the relationship between ASD severity and problem behavior was not significant, *b* = .16, *t* = .86, *p* = .40 [−.22, .53]; rather, the degree of problem behavior was high at all severities of ASD. The Johnson–Neyman technique revealed a zone of significance from a *T*-score of 44.00 (lower bound of data) to a *T*-score of 62.15. The relationship between ASD severity and problem behavior was no longer significant for individuals in the sample with a *T*-score above 62.15.

## Discussion

This study aimed to examine the influence of accompanying sleep disturbance on the established relationship between ASD symptom severity and problem behavior in ASD. Although a significant literature exists on the relationships between ASD severity, sleep disturbance, and problem behavior, relatively little is known about whether ASD severity is related to problem behavior comparably across individuals with varying degrees of accompanying sleep disturbance (i.e., none, mild, and moderate to severe). The current study employed a series of moderation analyses to explore this and revealed that the relationship between ASD severity and problem behavior varied significantly depending on the degree of accompanying sleep disturbance present. For individuals with no sleep disturbance or mild sleep disturbance, ASD symptom severity and problem behavior were positively related. For these individuals, having milder ASD symptoms was associated with significantly fewer problem behaviors; it was only those with the most severe ASD symptoms who experienced problem behavior that reached clinical levels. In contrast, ASD symptom severity and problem behavior were not related in individuals with moderate-to-severe sleep disturbance; rather, these individuals exhibited clinically significant problem behavior regardless of whether they had mild, moderate, or severe ASD symptoms. This suggests that individuals with moderate-to-severe sleep disturbance in addition to an ASD, of any severity, are likely to experience problem behavior at levels high enough to warrant clinical attention. This is in contrast to those with milder sleep disturbance or no sleep disturbance who appear incrementally more likely to experience clinically significant problem behavior as ASD symptom severity increases.

These findings complement the broader literature in this area and, importantly, offer novel information regarding the complex interaction between ASD symptom severity and accompanying sleep disturbance. Many researchers have previously acknowledged that there is a relationship between ASD symptom severity and problem behavior (24, 35–38), and while our results mirror this argument in individuals with no significant sleep disturbance and milder sleep disturbance, we did not find evidence of this relationship for those with moderate-to-severe sleep disturbance. This revelation is novel and emphasizes that the relationship between ASD symptom severity and problem behavior may be more nuanced than previously described.

To extend our findings and gain further insight into whether specific types of sleep problems influence the relationship between ASD symptom severity and problem behavior, we conducted a series of follow-up moderation analyses, considering each sleep domain in turn: bedtime resistance, sleep onset delay, sleep duration, sleep anxiety, night waking, parasomnias, disordered breathing, and daytime sleepiness. Some sleep domains revealed a similar pattern of results to the overall sleep model, whereby the relationship between ASD symptom severity and problem behavior was only apparent for those with no sleep problems or milder sleep problems, whereas other domains revealed that the relationship between ASD symptom severity and problem behavior was similar across all levels of sleep disturbance (i.e., none, mild, and moderate to severe). More specifically, there was evidence that ASD symptom severity and degree of sleep disturbance in each of the following areas—bedtime resistance, sleep anxiety, night waking, and parasomnias—individually predicted level of problem behavior. The relationships were all positive and indicated that individuals with more severe ASD symptoms or a higher degree of sleep disturbance in these specific domains were likely to experience greater problem behaviors. There was no evidence of moderation in these domains, which suggests that having milder ASD may be of some benefit when sleep problems manifest as bedtime resistance, sleep anxiety, night waking, and parasomnias.

Sleep onset delay showed a similar pattern to domains described above; however, for this domain, only ASD symptom severity predicted problem behavior. The severity of sleep onset delay itself did not reach significance, nor was there any evidence of moderation. One consideration here is that delayed sleep can occur for a variety of reasons, which may affect the overall quantity and quality of sleep to different degrees. Sleep latency may be extended as a consequence of other problems such as bedtime resistance, sleep anxiety, or sleep associations (18), and when evaluating how problematic delayed sleep may be for an individual, it is important to consider not only the cause but also whether sleep is adequately maintained after onset and whether overall duration is sufficient.

The results from the remaining three sleep domains revealed sleep duration, disordered breathing, and daytime sleepiness as significant moderators of the relationship between ASD symptom severity and problem behavior. Unlike the total sleep score on the CSHQ, means and standard deviations are available for the individual domains, and thus conversion to *T*-scores was performed for ease of interpretation (see Measures section for further details). For sleep duration, disordered breathing, and daytime sleepiness, the relationships between ASD severity and problem behavior were positive and significant up to a *T*-score of 65, 64, and 62 on the sleep scales, respectively.^1^ When the degree of sleep disturbance in these domains exceeded these *T*-scores, the relationship between ASD severity and problem behavior was no longer significant. Notably, each of these values is above normative limits and approaching clinical significance. These findings suggest that when problems with sleep duration, disordered breathing, and daytime sleepiness reach more significant levels, ASD symptom severity appears less relevant. While there was also some evidence of significance at very high levels of disordered breathing (*T*-scores exceeding 117), further investigation is required to examine the nature of this relationship given that relatively few participants scored in this range in the current cohort.

As per the broader results, the domain analyses both complement and extend the existing literature in this area. In particular, the bedtime resistance, sleep anxiety, night waking, and parasomnias results align closely with studies that have presented ASD severity (24, 35–38) or accompanying sleep disturbance (23, 25, 27, 29, 33) as key factors influencing problem behavior. They also align closely with studies that have indicated a relationship between severity of sleep disturbance and degree of problem behavior [e.g., Refs. (28, 29)]. As for sleep duration, disordered breathing, and daytime sleepiness, the results do not necessarily contradict the existing literature, but they do clarify that the association typically observed between ASD symptom severity and problem behavior varies as a function of how severe an individual’s difficulties are with sleep duration, disordered breathing, or daytime sleepiness. They further clarify that high levels of these specific sleep problems are associated with clinically significant problem behavior even in individuals with milder ASD. Research into the types of sleep disturbance that may be most important to consider in the context of problem behavior is still being investigated. While some connection can be drawn between the domains that emerged in this study and those of existing studies [e.g., Refs. (15, 19, 34)], direct comparison and further progress are limited by the way in which researchers conceptualize and measure sleep disturbance. There appears to be little consensus among methods of determining severity and significant overlap in the breakdown of sleep domains captured both within and between different measures.

While the results and implications of this study are novel and clinically relevant, they do need to be interpreted with some degree of caution until they are able to be replicated. The study is limited by its relatively small sample size and large age span. Due to recruitment methods, and the nature of the broader study, the sample was exclusively male, which, on the one hand, may be considered a strength given the frequent differences in abilities and behaviors observed between males and females with ASD but, on the other hand, limits generalizability. This factor also limited how much information we were able obtain around medications and interventions that may have been prescribed to help manage sleep disturbance in the sample. The potential influence of these factors is important to consider and should be examined in future related research wherever feasible. It is also important to note that while ASD symptom severity and accompanying sleep disturbance are important factors that appear to explain a very significant portion of variance, they are not the only factors that have the potential to influence problem behavior. Another broad consideration to keep in mind with research of this nature is that the relationships between these variables are complex and unlikely to be unidirectional. While the rhetoric developed in this paper focused on the effect that ASD symptom severity and sleep disturbance may have on problem behavior, there is also literature to indicate that sleep disturbance may elevate ASD symptom severity (23, 39–41) and a possibility that problem behavior may exacerbate difficulties with sleep. Future research aimed at capturing the broad range of (dis)abilities across the spectrum will enable us to further our understanding of how core factors and comorbidities affect different individuals and may improve our ability to predict outcomes and long-term functioning. We suggest sleep as a key avenue to continue this endeavor, particularly given that problems are potentially treatable with behavioral sleep interventions, light therapy, and/or pharmacological options, and when identified for treatment can have reaching, positive consequences for an individual’s functioning [e.g., Ref. (48)].

In sum, key findings from this study indicated a positive relationship between ASD severity and problem behavior for individuals with no sleep disturbance or milder sleep disturbance, yet no significant relationship between ASD symptom severity and problem behavior for those with moderate-to-severe sleep disturbance. Clinically significant problem behavior was apparent across all individuals with moderate-to-severe sleep disturbance regardless of their ASD symptom severity, and this pattern was particularly apparent for sleep duration, disordered breathing, and daytime sleepiness. Overall, the findings highlight the variability among individuals with ASD and demonstrate that profiling individuals on the basis of their sleep habits may help to identify those who are most likely to experience clinically significant problem behavior. More broadly, the findings also seem to suggest that those with one clinically significant issue (i.e., sleep disturbance) may be more vulnerable to other clinically significant issues or comorbid conditions (e.g., psychopathologies such as anxiety and depression) and reinforce the need for careful assessment and early intervention.

## Ethics Statement

This study was carried out in accordance with the recommendations of the Deakin University Human Research Ethics Committee (DUHREC) and the Victorian Department of Education and Training with written informed consent from all subjects. All subjects gave written informed consent in accordance with the Declaration of Helsinki. The protocol was approved by DUHREC.

## Author Contributions

All authors, EL, CS, TM, NS, KH and NR, participated in the conception and design of the study. EL, CS, KH, and NS were involved in recruitment and data collection. EL and NS analyzed the data. All authors were involved in data interpretation and manuscript drafting. All authors read and approved the final manuscript.

## Funding

The broader longitudinal study of which this work forms a part was supported by philanthropic donation from Moose Toys.

## Conflict of Interest Statement

NR currently receives funding from the Moose Foundation, Victorian Department of Education and Training, MECCA Brands, Wenig Family, Geelong Community Foundation, and Grace & Emilio Foundation to conduct research in the field of neurodevelopmental disorders and inclusion. NR also receives funding from the Ferrero Group Australia as part of its Kinder + Sport pillar of Corporate Social Responsibility initiatives to promote active lifestyles among young people. NR has previously received donations from Vic Health and Bus Association Victoria; received previous speaker honorarium from Novartis (2002), Pfizer (2006), and Nutricia (2007); and is a Director of the Amaze Board (Autism Victoria). None of the companies or organizational bodies listed above had a role in this research including the collection, analysis, and interpretation of data; in writing of the manuscript; and/or in the decision to submit the article for publication.

The remaining authors declare that the research was conducted in the absence of any commercial or financial relationships that could be construed as a potential conflict of interest.

## Footnotes

1 As previously indicated, generally, T-scores between 60 and 64 are considered higher than average and may indicate slightly more concerns than is typical; those between 65 and 69 are notably elevated and border clinical significance, while those above 70 warrant clinical concern.

## References

[1] APA (2013). Diagnostic and statistical manual of mental disorders. Washington, DC: American Psychiatric Publishing.

[2] Schroeder J Weiss J Bebko J CBCL profiles of children and adolescents with Asperger syndrome: a review and pilot study. J Dev Disabil (2011) 17(1):26–37. http://oadd.org/wp-content/uploads/2011/01/41009_JoDD_17-1_26-37_Schroeder_et_al.pdf

[3] Hartley SL Sikora DM McCoy R Prevalence and risk factors of maladaptive behaviour in young children with autistic disorder. J Intellect Disabil Res (2008) 52(10):819–29. 10.1111/j.1365-2788.2008.01065.x PMC2838711 18444989

[4] Solomon M Ozonoff S Carter C Caplan R Formal thought disorder and the autism spectrum: relationship with symptoms, executive control, and anxiety. J Autism Dev Disord (2008) 38(8):1474–84. 10.1007/s10803-007-0526-6 PMC5519298 18297385

[5] Bauminger N Solomon M Rogers SJ Externalizing and internalizing behaviors in ASD. Autism Res (2010) 3(3):101–12. 10.1002/aur.131 PMC5659193 20575109

[6] Chandler S Howlin P Simonoff E O’Sullivan T Tseng E Kennedy J Emotional and behavioural problems in young children with autism spectrum disorder. Dev Med Child Neurol (2016) 58(2):202–8. 10.1111/dmcn.12830 26077499

[7] Chen C Shen YD Xun GL Cai WX Shi LJ Xiao L Aggressive behaviors and treatable risk factors of preschool children with autism spectrum disorder. Autism Res (2017) 10(6):1155–62. 10.1002/aur.1751 28266803

[8] McClain MB Hasty Mills AM Murphy LE Inattention and hyperactivity/impulsivity among children with attention-deficit/hyperactivity-disorder, autism spectrum disorder, and intellectual disability. Res Dev Disabil (2017) 70:175–84. 10.1016/j.ridd.2017.09.009 28957735

[9] Summers J Shahrami A Cali S D’Mello C Kako M Palikucin-Reljin A Self-injury in autism spectrum disorder and intellectual disability: exploring the role of reactivity to pain and sensory input. Brain Sci (2017) 7(11):1–16. 10.3390/brainsci7110140 PMC5704147 29072583

[10] Tonge B Brereton A Gray KM EInfield SL Behavioural and emotional disturbance in high-functioning autism and Asperger syndrome. Autism (1999) 3(2):117–30. 10.1177/1362361399003002003

[11] Wijnhoven L Creemers DHM Vermulst AA Granic I Prevalence and risk factors of anxiety in a clinical Dutch sample of children with an autism spectrum disorder. Front Psychiatry (2018) 9:1–10. 10.3389/fpsyt.2018.00050 29551982 PMC5840159

[12] Bub KL McCartney K Willett JB Behavior problem trajectories and first-grade cognitive ability and achievement skills: a latent growth curve analysis. J Educ Psychol (2007) 99(3):653–70. 10.1037/0022-0663.99.3.653

[13] Langstrom N Grann M Ruchkin V Sjostedt G Fazel S Risk factors for violent offending in autism spectrum disorder: a national study of hospitalized individuals. J Interpers Violence (2009) 24(8):1358–70. 10.1177/0886260508322195 18701743

[14] Cohen S Conduit R Lockley SW Rajaratnam SM Cornish KM The relationship between sleep and behavior in autism spectrum disorder (ASD): a review. J Neurodev Disord (2014) 6(44):1–10. 10.1186/1866-1955-6-44 25530819 PMC4271434

[15] Mazurek MO Sohl K Sleep and behavioral problems in children with autism spectrum disorder. J Autism Dev Disord (2016) 46(6):1906–15. 10.1007/s10803-016-2723-7 26823076

[16] Stickgold R A memory boost while you sleep. Neuroscience (2006) 444:559–60. 10.1038/nature05309 17086196

[17] Graven SN Browne JV Sleep and brain development: the critical role of sleep in fetal and early neonatal brain development. Newborn Infant Nurs Rev (2008) 8(4):173–9. 10.1053/j.nainr.2008.10.008

[18] Cortesi F Giannotti F Ivanenko A Johnson K Sleep in children with autistic spectrum disorder. Sleep Med (2010) 11(7):659–64. 10.1016/j.sleep.2010.01.010 20605110

[19] Hirata I Mohri I Kato-Nishimura K Tachibana M Kuwada A Kagitani-Shimono K Sleep problems are more frequent and associated with problematic behaviors in preschoolers with autism spectrum disorder. Res Dev Disabil (2016) 49–50:86–99. 10.1016/j.ridd.2015.11.002 26672680

[20] Mazzone L Postorino V Siracusano M Riccioni A Curatolo P The relationship between sleep problems, neurobiological alterations, core symptoms of autism spectrum disorder, and psychiatric comorbidities. J Clin Med (2018) 7(5):1–12. 10.3390/jcm7050102 PMC5977141 29751511

[21] Hollway JA Aman MG Sleep correlates of pervasive developmental disorders: a review of the literature. Res Dev Disabil (2011) 32(5):1399–421. 10.1016/j.ridd.2011.04.001 21570809

[22] Patzold LM Richdale AL Tonge BJ An investigation into sleep characteristics of children with autism and Asperger’s disorder. J Paediatr Child Health (1998) 34:528–33. 10.1046/j.1440-1754.1998.00291.x 9928644

[23] Goldman SE Surdyka K Cuevas R Adkins K Wang L Malow BA Defining the sleep phenotype in children with autism. Dev Neuropsychol (2009) 34(5):560–73. 10.1080/87565640903133509 PMC2946240 20183719

[24] Mayes SD Calhoun SL Variables related to sleep problems in children with autism. Res Autism Spectr Disord (2009) 3(4):931–41. 10.1016/j.rasd.2009.04.002

[25] Goldman SE McGrew S Johnson KP Richdale AL Clemons T Malow BA Sleesp is associated with problem behaviors in children and adolescents with autism spectrum disorders. Res Autism Spectr Disord (2011) 5(3):1223–9. 10.1016/j.rasd.2011.01.010

[26] Henderson JA Barry TD Bader SH Jordan SS The relation among sleep, routines, and externalizing behavior in children with an autism spectrum disorder. Res Autism Spectr Disord (2011) 5(2):758–67. 10.1016/j.rasd.2010.09.003

[27] Park S Cho S-C Cho IH Kim B-N Kim J-W Shin M-S Sleep problems and their correlates and comorbid psychopathology of children with autism spectrum disorders. Res Autism Spectr Disord (2012) 6(3):1068–72. 10.1016/j.rasd.2012.02.004

[28] Sikora DM Johnson K Clemons T Katz T The relationship between sleep problems and daytime behavior in children of different ages with autism spectrum disorders. Pediatrics (2012) 130(Suppl 2):S83–S90. 10.1542/peds.2012-0900F 23118258

[29] Adams HL Matson JL Jang J The relationship between sleep problems and challenging behavior among children and adolescents with autism spectrum disorder. Res Autism Spectr Disord (2014) 8(9):1024–30. 10.1016/j.rasd.2014.05.008

[30] Nadeau JM Arnold EB Keene AC Collier AB Lewin AB Murphy TK Frequency and clinical correlates of sleep-related problems among anxious youth with autism spectrum disorders. Child Psychiatry Hum Dev (2015) 46(4):558–66. 10.1007/s10578-014-0496-9 25239284

[31] Cohen S Fulcher BD Rajaratnam SMW Conduit R Sullivan JP St Hilaire MA Sleep patterns predictive of daytime challenging behavior in individuals with low-functioning autism. Autism Res (2018) 11(2):391–403. 10.1002/aur.1899 29197172

[32] May T Cornish K Conduit R Rajaratnam SM Rinehart NJ Sleep in high-functioning children with autism: longitudinal developmental change and associations with behavior problems. Behav Sleep Med (2013) 13(1):2–18. 10.1080/15402002.2013.829064 24283751

[33] Malow BA Marzec ML McGrew SG Wang L Henderson LM Stone WL Characterizing sleep in children with autism spectrum disorders: a multidimensional approach. Sleep (2006) 29(12):1563–71. 10.1093/sleep/29.12.1563 17252887

[34] Fadini CC Lamonica DA Fett-Conte AC Osorio E Zuculo GM Giacheti CM Influence of sleep disorders on the behavior of individuals with autism spectrum disorder. Front Hum Neurosci (2015) 9:1–8. 10.3389/fnhum.2015.00347 26150777 PMC4471742

[35] Matson JL Wilkins J Macken J The relationship of challenging behaviors to severity and symptoms of autism spectrum disorders. J Ment Health Res Intellect Disabil (2008) 2(1):29–44. 10.1080/19315860802611415

[36] Jang J Dixon DR Tarbox J Granpeesheh D Symptom severity and challenging behavior in children with ASD. Res Autism Spectr Disord (2011) 5(3):1028–32. 10.1016/j.rasd.2010.11.008

[37] Tudor ME Hoffman CD Sweeney DP Children with autism: sleep problems and symptom severity. Focus Autism Other Dev Disabl (2012) 27(4):254–62. 10.1177/1088357612457989

[38] Andersen PN Hovik KT Skogli EW Oie MG Severity of autism symptoms and degree of attentional difficulties predicts emotional and behavioral problems in children with high-functioning autism: a two-year follow-up study. Front Psychol (2017) 8:1–9. 10.3389/fpsyg.2017.02004 29184527 PMC5694568

[39] Schreck K Sleep problems as possible predictors of intensified symptoms of autism. Res Dev Disabil (2004) 25(1):57–66. 10.1016/j.ridd.2003.04.007 14733976

[40] Hundley RJ Shui A Malow BA Relationship between subtypes of restricted and repetitive behaviors and sleep disturbance in autism spectrum disorder. J Autism Dev Disord (2016) 46(11):3448–57. 10.1007/s10803-016-2884-4 27511195

[41] Thenhausen N Kuss M Wiater A Schlarb AA Sleep problems in adolescents with Asperger syndrome or high-functioning autism. Somnologie (2016) 21(S1):28–36. 10.1007/s11818-016-0078-0

[42] Sparrow SS Cicchetti DV Saulnier CA Vineland adaptive behavior scales, third edition (Vineland -3). Bloomington: NCS Pearson (2016).

[43] Constantino JN Gruber CP Social responsiveness scale—second edition (SRS-2). Torrance, CA: Western Psychological Services (2012).

[44] Owens JA Spirito A McGuinn M The children’s sleep habits questionnaire (CSHQ): psychometric properties of a survey instrument for school-aged children. Sleep (2000) 23(8):1–9. 10.1093/sleep/23.8.1d 11145319

[45] Achenbach TM Rescorla LA Manual for the ASEBA preschool forms & profiles. Burlington, VT: University of Vermont, Research Center for Children, Youth, & Families (2000).

[46] Achenbach TM Rescorla LA (2001). Manual for the ASEBA School-Age Forms & Profiles. Burlington, VT: University of Vermont, Research Center for Children, Youth, & Families.

[47] Hayes AF (2013). Introduction to mediation, moderation, and conditional process analysis: a regression-based approach. New York: The Guildford Press.

[48] Papadopoulos N Sciberras E Hiscock H Mulraney M McGillivray J Rinehart N The efficacy of a brief behavioral sleep intervention in school-aged children with ADHD and comorbid autism spectrum disorder. J Atten Disord (2019), 23(4):341–50. 10.1177/1087054714568565 25646022

